# Investigating Effect of Rapamycin and Metformin on Angiogenesis in Hepatocellular Carcinoma Cell Line

**DOI:** 10.15171/apb.2018.008

**Published:** 2018-02-18

**Authors:** Mandana Rastegar, Haji-Amin Marjani, Yaghoub Yazdani, Majid Shahbazi, Masoud Golalipour, Touraj Farazmandfar

**Affiliations:** Medical Cellular and Molecular Research Center, Golestan University of Medical Sciences, Gorgan, Iran.

**Keywords:** Metformin, Rapamycin, Hepatocellular carcinoma

## Abstract

***Purpose:*** Human hepatocellular carcinoma is one of the most common causes of death in the world. Metformin and rapamycin may decrease the expression of VEGF protein and subsequently angiogenesis. The purpose of this study was to evaluate the effect of these two drugs on expression of VEGF protein and the cell proliferation in the hepatocellular carcinoma cell line (ATCC HB-8065).

***Methods:*** HepG2 was cultured in RPMI-1640 medium at 37°C for 48h as a pre-culture and then treated by different concentrations of metformin (0, 5, 10 and 20 mM) and rapamycin (0, 5, 10 and 20 nM) at different times (12, 24 and 48 h). Cell viability was assessed by the MTT assay. Total RNA was extracted by the Trizol reagent and VEGF gene expression was analyzed by quantitative real-time PCR and was calculated by 2^–ΔCt^ method. The VEGF protein level was determined by Elisa assay. Finally, Apoptosis index was calculated by DAPI staining.

***Results:*** Metformin and rapamycin significantly decrease cancer cells viability (p<0.05). Rapamycin but not metformin decreases VEGF gene expression in HepG2 cells. Metformin and rapamycin significantly induce cell apoptosis in hepatocellular carcinoma (HCC) cells.

***Conclusion:*** Metformin and rapamycin have an anti-tumor effect on HCC. According to our data rapamycin might have an anti-angiogenesis effect via inhibition of VEGF expression. Our results provide an insight into future clinical strategies to improve chemotherapy outcomes in HCC.

## Introduction

Hepatocellular carcinoma (HCC) is the most common primary malignant tumor of the liver and is the fourth leading cause of cancer-related death in the world.^1^ HCC is seventh most common cancer in men and the ninth in women.^2^ However, the increased prevalence of HCC in communities with a high risk, in men is higher than women.^3^ HCC is an aggressive malignancy with poor prognosis. Current chemotherapeutic drugs are not effective to control tumor growth and drug resistance is a common problem in a successful therapy. Therefore, novel approaches in chemotherapy may be needed to improve the survival rate in patients with HCC.^4^ The treatment with anti-angiogenic agents may be a promising approach to treat HCC. Angiogenesis is a complex process that is based on cooperation between various cells, such as pericytes, endothelial cells, fibroblasts and smooth muscle cells.‏ These cells produce a variety of cytokines and growth factors that interact with other cells or with the extracellular matrix and affect migration, proliferation and angiogenesis.^5^ Angiogenesis plays a key role in tumor growth, progression and metastasis. This uncontrolled growth is well established in many tumors such as ovarian,^6^ lung,^7^ colon,^8^ prostate,^9^ brain^10^ and lymphoid^11^ tumors. Inhibition of angiogenesis may be a valuable method for controlling cancer. Without angiogenesis, tumor growth potentially is limited due to lack of blood flow and proliferation factors. Metastatic potential of tumors develops through the increased angiogenesis. This process was performed by disrupting the balance between of pro-angiogenic and anti-angiogenic factors via increasing the presence of pro-angiogenic factors such as VEGF.^12^ Some studies on angiogenesis inhibitors in animal models showed that angiogenesis inhibition can destroy many types of tumors.^13^ Two strategies used in the development of anti-angiogenic agents include exogenous angiogenic inhibitors such as monoclonal antibody against VEGF, and endogenous inhibitors such as endostatin and angiostatin.^14^

Metformin is one of the oldest and most commonly used drugs in the treatment of type 2 diabetes that reduces blood sugar by decreasing insulin resistance, with no effect on insulin secretion.^15^ Serious side effects of this drug are rare and mostly due to the simultaneous presence of other diseases in diabetic patients, and not by the drug itself.^16^ Recently, several studies have shown that metformin may reduce the risk of liver cancer.^17-19^ Metformin interfere with glucose metabolism that leads to changes in the intracellular pathways. One of these changes is the increased expression of the enzyme 5' adenosine monophosphate-activated protein kinase (AMPK) in liver cells by which the mammalian target of rapamycin (mTOR) protein was inhibited.^20^ It is known that increased expression of the protein mTOR leads to the increased expression of the transcription factor hypoxia-inducible factor 1-alpha (HIF1α) and this factor increases the intensity of vessel endothelial growth factor (VEGF).^21^ Therefore, metformin indirectly increase VEGF. VEGF is a 45 kDa glycoprotein that increases angiogenesis and vascular endothelial cell proliferation. VEGF is one of the strongest and most important angiogenic factors in the body. VEGF is one of the most important angiogenesis factors that was overexpressed in many cancers.^22^

Rapamycin is known as an antibiotic and with a mechanism similar to metformin, inhibits mTOR and ultimately reduce VEGF expression. This drug is used as an immune suppressor to prevent organ rejection after liver transplantation.^23^ Recently, several studies have shown that rapamycin in combination with other medicines reduced proliferation of HCC cells in vitro and animal models.^24,25^

Considering above evidences it can be concluded that drugs of metformin and rapamycin may indirectly reduce the expression of VEGF and subsequently decrease angiogenesis in cancerous tissues. Because the major effects of these drugs on liver cells, in this study, we evaluated the effect of these two drugs on VEGF expression and the cell proliferation in the liver carcinoma cell line.

## Materials and Methods

### Cell culture

The Human liver cancer cell line, HepG2 (ATCC HB-8065, Manassas, USA) was cultured in RPMI-1640 medium (Thermo Fisher Scientific, Massachusetts, USA), contained 10% fetal bovine serum (Invitrogen, Darmstadt, Germany) and 100 U/mL penicillin/streptomycin (Invitrogen, Darmstadt, Germany) in a CO2 incubator at 37°C for 48h as a pre-culture. The treatment program included concentrations of metformin (Sigma, Munich, Germany) (0, 5, 10 and 20mM) and rapamycin (Sigma, Munich, Germany) (0, 5, 10 and 20nM) at different times (12, 24 and 48 h).

### MTT assay 

Cell viability after drug treatment was investigated by a standard colorimetric assay using 3-(4 5-dimethylthiazol-2-yl)-2 5-diphenyltetrazolium bromide (MTT) reagent (Sigma, Munich, Germany. First, 5,000 cells were seeded in 100 μl medium in a 96-well plate (Thermo Fisher Scientific, Massachusetts, USA) and incubated at 37°C with 5 % CO2 for 24 hours before adding drugs to allow cell adhesion. Then the medium was removed and cells were washed with 50 μl Phosphate-buffered saline (PBS) that was then removed. Following cells were treated in triple tests with the different concentrations (0, 5, 10 and 20 mM of metformin and 0, 5, 10 and 20 nM of rapamycin) at different times (12, 24 and 48 h). 50 μl MTT solution (5 mg/ml in PBS) was added to each well and was incubated for 3 h at 37°C. The optical density was then measured at 570 nm using ELISA Plate Readers (BioTek, Winooski, USA).

### Quantitative real-time PCR

Total RNA was extracted by the Trizol reagent (Thermo Fisher Scientific, Massachusetts, USA) according to manufacturer’s protocol. First-strand complementary DNA (cDNA) was synthesized using SuperScript III First-Strand Synthesis System (Thermo Fisher Scientific, Massachusetts, USA) according to the manufacturer’s instrument. The specific primers for VEGF were forward 5′-GGGCACTGCCTGGAAGATTCAG-3′ and reverse 5′-CTTCTCTTCGCCGGGACATCTG-3′, and for Glyceraldehyde 3-phosphate dehydrogenase (GAPDH) were forward 5′-GGTGGTCTCCTCTGACTTCAACA-3′ and reverse 5′-GTTGCTGTAGCCAAATTCGTTGT-3′, which were designed by GeneRunner software (version 5; Hastings, USA) and were reviewed in BLAST websites. Quantitative Real-time PCR was performed in triple tests in a detection System (Line-Gene K, BIOER, Hangzhou, China) by a real-time PCR kit (Thermo Fisher, Massachusetts, USA) as previously described.^26^ PCR conditions started at one step initial denaturation (95°C for 3 min), followed by 40 cycles (95°C for 20 sec, 60°C for 20 sec, and 72°C for 20 sec). VEGF and GAPDH mRNA level were expressed as the cycle threshold (Ct) values. VEGF mRNA level normalized against GAPDH mRNA level and was calculated by 2^–ΔCt^ method.^27^

### Elisa assay

To determine VEGF protein level, 5×10^3^ HepG2 cells were seeded in a 96-well plate and were treated with the different concentrations at different times. VEGF concentrations were then measured by ELISA (Abcam, Cambridge, USA) for each well as previously described.^28^

### Apoptosis index calculation

HepG2 Cells were fixed by Paraformaldehyde (Sigma, Munich, Germany) and stained by 4′,6′-diamidino-2-phenylindole dihydrochloride (DAPI) reagent (Sigma, Munich, Germany) according to the manufacturer’s protocol. Cells were then investigated under a fluorescent microscope (Nikon, Shanghai, China) at a 100x magnification. Cells were counted in 10 randomized selected microscopic fields in which apoptotic cells were characterized by condensation, nuclear shrinkage and fragmentation. The apoptosis index (AI) percentage was calculated using the following formula: AI (%) = (apoptotic cells/total cells) × 100%.^4^

### Statistical analysis

The experiment Data are expressed as the mean ± standard deviation. Data were analyzed using GraphPad software (version 6; San Diego, CA, United States). The difference between variables was performed by Student’s t-test or one-way ANOVA test. A P value less than 0.05 was considered statistically significant.

## Results and Discussion

### Metformin and rapamycin decrease cancer cells viability

**Figure 1:**
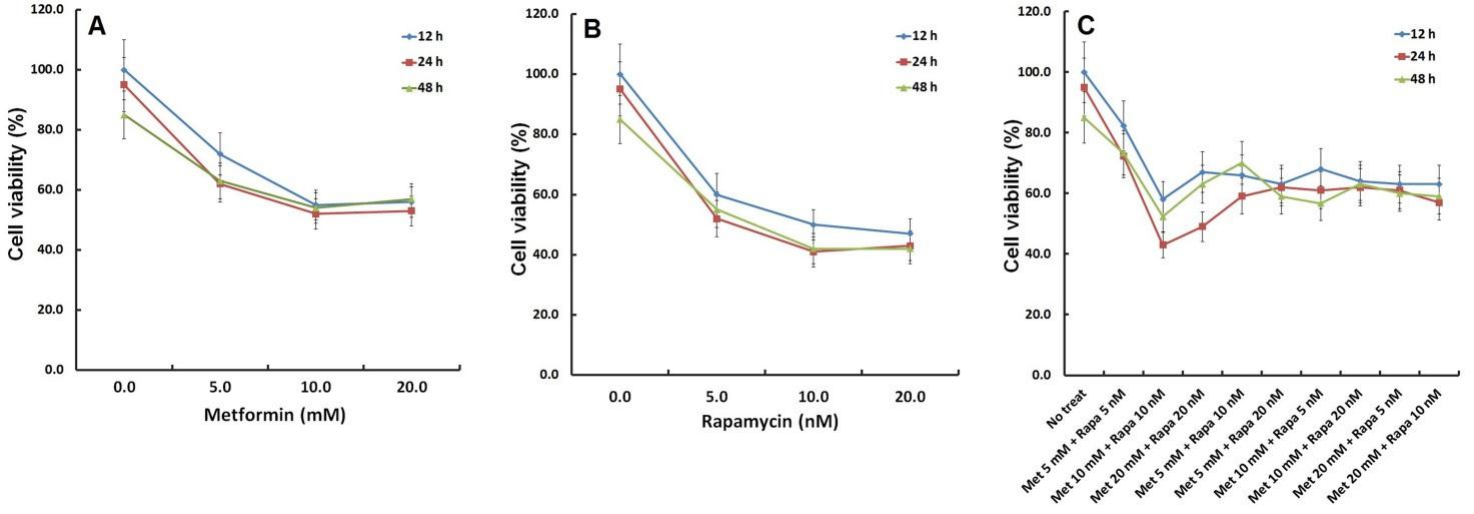
MTT assay for analyzing HepG2 cells viability after treatment with metformin and rapamycin.** (A)** Metformin has reduced hepatocarcinoma cells survival by about 50% in concentration 10 mM. **(B)** Rapamycin has reduced cells survival by about 60% in concentration 10mM. **(C)** The lowest survival is associated with cells treated by the combination of metformin 10mM and rapamycin 10 nM.

To evaluate the effects of metformin and rapamycin on HCC cells growth in vitro, cell viability of HepG2 Cells was assessed by MTT assay following treatment with metformin and rapamycin in various concentrations and at different times. MTT data showed that mean IC50 values from 3 independent experiments in times of 12, 24 and 48 h were 2.63 ± 0.52, 3.77 ± 0.69 and 3.74 ± 0.71 for metformin, and 3.83 ± 0.82, 3.54 ± 0.85 and 4.14 ± 0.91 for rapamycin. Results showed that both metformin and rapamycin inhibited the growth of HepG2 cells in a dose-dependent manner (p < 0.05) (Figure 1). It was also found that the lowest cell viability was seen in concentrations of metformin (10 mM) (Figure 1A) and rapamycin (10 nM) in 24 hours after treatment (Figure 1B). In addition, it was found that in combination with different concentrations of the two drugs, lowest survival cell viability was seen also in concentrations of metformin 10mM and rapamycin 10nM in 24 hours after treatment (Figure 1C).

### Rapamycin but not metformin decreases VEGF gene expression in HepG2 cells

**Figure 2:**
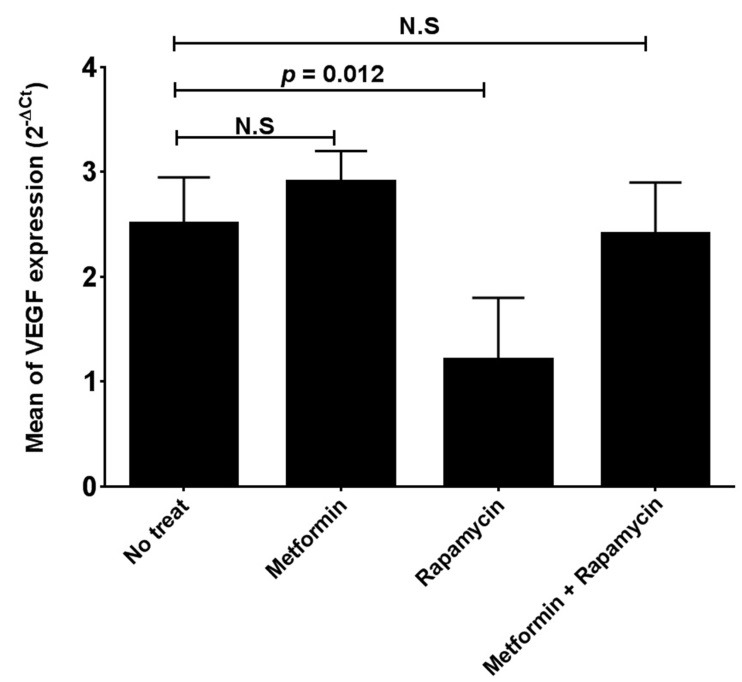
Quantitative real-time PCR for investigation of *VEGF* gene expression in HepG2 cells. Rapamycin but no metformin, significantly reduces the *VEGF* gene expression in the treated HepG2 cells in comparison with untreated cells. Metformin and rapamycin were used in concentrations of 10mM and 10nM respectively.

**Figure 3:**
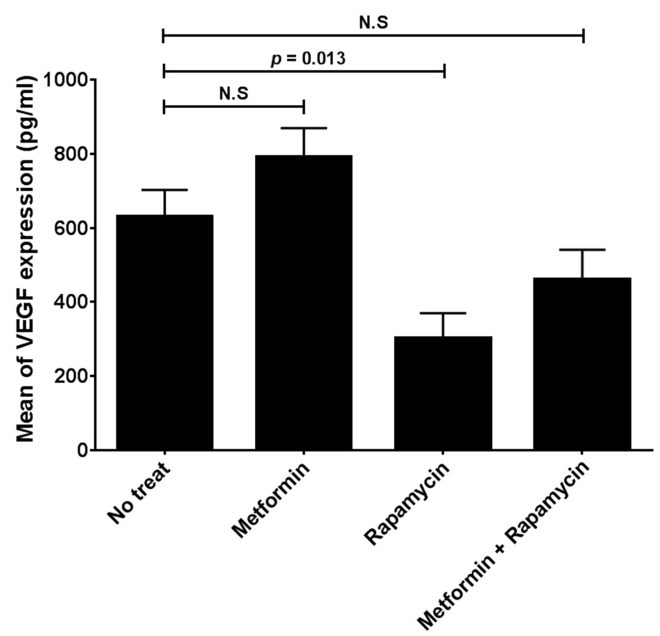
Elisa assay for determination of VEGF protein level in HepG2 cells. Rapamycin but no metformin significantly reduces the VEGF protein level in the treated HepG2 cells than untreated cells. Metformin and rapamycin were used in concentrations of 10mM and 10 nM respectively.

To study the inhibitory effects of metformin and rapamycin on HepG2 cells growth, we examined the expression of *VEGF* gene, in cells treated with metformin (10 mM), rapamycin (10 nM) and the combination of two drugs after 24 hours. Our results in Figure 2 showed that metformin has no significant effect on *VEGF* gene expression in the treated HepG2 cells compared to the untreated cells. In contrast, rapamycin significantly reduces the *VEGF* gene expression in HepG2 cell line in comparison with untreated cells (p = 0.012). The combination of two drugs has also no significant effect on *VEGF* gene expression in HepG2 cells (Figure 2). Moreover, to confirm VEGF expression analysis, we determined VEGF protein level in HepG2 cells treated with two drugs and the results are shown in Figure 3. These results showed that metformin has no significant effect on *VEGF* gene expression in treated HepG2 cells.‏ In contrast, Rapamycin significantly reduces the *VEGF* gene expression in HepG2 cells (p = 0.013). The combination of two drugs has also no significant effect on *VEGF* gene expression in treated HepG2 cells in comparison with untreated cells (Figure 3).

### Metformin and rapamycin induce cell apoptosis

**Figure 4:**
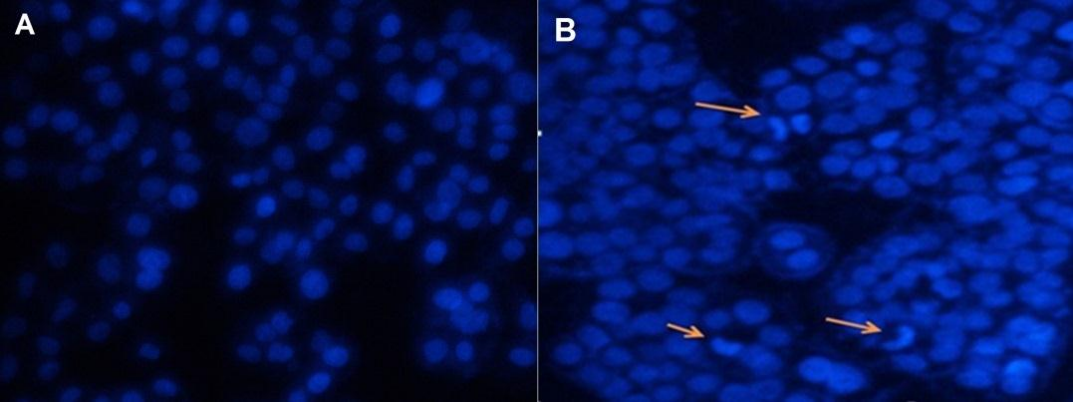
The nuclear morphological changes in HepG2 cells after treatment with metformin (10 mM) and rapamycin (10 nM). **(a)** The control cells showed intact nuclei with uniform shape and size. **(b)** Arrows indicate apoptotic bodies observed at 40X magnification under an inverted fluorescence microscope.

**Figure 5:**
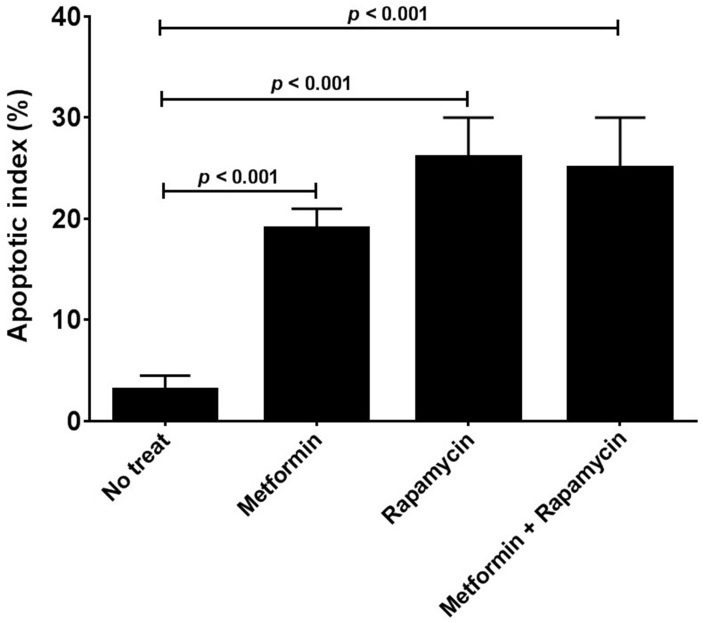
Apoptotic index analysis in the treated HepG2 cells with metformin (10 mM) and rapamycin (10 nM). Apoptosis index in the treated cells with both drugs (separately and in combination) was significantly high in comparison with untreated cells.

To investigate whether metformin and rapamycin induce cellular apoptosis, HepG2 cells were incubated with metformin (10mM), rapamycin (10nM) and the combination of two drugs at the same concentration. After 24 hours, nuclear morphology by DAPI staining was assessed (Figure 4). The results in Figure 5 showed that the apoptosis index in the treated cells with both drugs was significantly higher in comparison with untreated cells (p < 0.001).

Nowadays the use of VEGF pathway inhibitors in the angiogenesis is considered as a therapeutic strategy against cancer with a clinical validation.^12^ In this study, we suggested that these agents should be used in combination with other therapeutic agents.^29^ Therefore, further studies are needed to understand the effective combination therapies in reducing tumor angiogenesis and drug resistance. Despite significant advances in the control and treatment of malignant tumors, the search continues for new treatments and drugs. In this way, considering the molecular pathways involved in tumor growth and identify ways to control these pathways has attracted a more attention. The two drugs, which have been provided with specific molecular targets, are metformin and rapamycin. These two drugs indirectly inhibit expression of VEGF.^24^ Therefore; it was assumed that these two drugs lead to reduced VEGF expression and subsequently to inhibit angiogenesis in tumors. Our results in this study showed that rapamycin but not metformin inhibits *VEGF* gene expression in HepG2 cells. Different effects of metformin treatment on gene expression can be due to several factors as mentioned in the Dallaglio et al. study in which they observed a paradoxical effect of metformin on endothelial and cancerous cells in the control of angiogenesis. They also concluded that effect of metformin on VEGF expression in each cell might be different.^30^ These differences are probably due to the difference of cancerous cells in the resistance to a specific drug. The Miyoshi et al. in a study has shown that metformin inhibits angiogenesis in hepatocyte carcinoma cell line with no effect on VEGF expression.^31^ The results of cell viability assay showed that metformin and rapamycin decrease cancer cells viability in HepG2 cells. This may be due to indirectly decrease of VEGF and subsequent activation of the phosphorylation of extracellular signal regulated kinase 1 (ERK1) and ERK2 as previously described.^32^ In agreement with our study, the Wang et al. showed that rapamycin reduces VEGF expression. They found even a low dose of rapamycin is sufficient to prevent the progression of HCC tumors.^33^ According to this study, it can be concluded that metformin can inhibit angiogenesis, but in the regulation of *VEGF* gene expression may demonstrate a dual effect. This dual effect probably depends on factors such as the type of cell, the duration of treatment, the drug concentration and other different signaling pathways. Confusion in this study may be due to the effect of combining the two drugs on VEGF expression. It seems that VEGF expression level in cells treated with the combination of two drugs is approximately equivalent to the average of expression level in cells treated with either drug separately. Although more comprehensive studies are needed to clarify it.

## Conclusion

The differential effect of metformin and rapamycin on VEGF expression calls into question the use of mTOR inhibitors to target angiogenesis. Further analysis of the molecular pathways affected by both drugs may lead the authors to define new therapeutic strategies. This information may help to design a combination treatment to increase the efficacy of metformin and rapamycin in HCC.

## Acknowledgments

This work was supported by the research department in Golestan University of Medical Sciences, Gorgan, Iran (Grant number 930705044).

## Ethical Issues

Not applicable.

## Conflict of Interest

The authors declare no conflict of interests.

## References

[1] Tsochatzis EA, Meyer T, Burroughs AK (2012). Hepatocellular carcinoma. N Engl J Med.

[2] Zhu RX, Seto WK, Lai CL, Yuen MF (2016). Epidemiology of Hepatocellular Carcinoma in the Asia-Pacific Region. Gut Liver.

[3] Wang Z, Xiang Q, Li D, Li S (1991). Correlation between gene expression and chromatin conformation of c-fos and N-ras in human liver and hepatoma. Chin Med Sci J.

[4] Qu Z, Zhang Y, Liao M, Chen Y, Zhao J, Pan Y (2012). In vitro and in vivo antitumoral action of metformin on hepatocellular carcinoma. Hepatol Res.

[5] Taghizadeh S, Sankian M, Ajami A, Tehrani M, Hafezi N, Mohammadian R (2014). Expression Levels of Vascular Endothelial Growth Factors A and C in Patients with Peptic Ulcers and Gastric Cancer. J Gastric Cancer.

[6] Shaw D, Clamp A, Jayson GC (2013). Angiogenesis as a target for the treatment of ovarian cancer. Curr Opin Oncol.

[7] Hall RD, Le TM, Haggstrom DE, Gentzler RD (2015). Angiogenesis inhibition as a therapeutic strategy in non-small cell lung cancer (NSCLC). Transl Lung Cancer Res.

[8] Sun W (2012). Angiogenesis in metastatic colorectal cancer and the benefits of targeted therapy. J Hematol Oncol.

[9] Russo G, Mischi M, Scheepens W, De la Rosette JJ, Wijkstra H (2012). Angiogenesis in prostate cancer: onset, progression and imaging. BJU Int.

[10] Jain RK, di Tomaso E, Duda DG, Loeffler JS, Sorensen AG, Batchelor TT (2007). Angiogenesis in brain tumours. Nat Rev Neurosci.

[11] Ribatti D, Nico B, Ranieri G, Specchia G, Vacca A (2013). The Role of Angiogenesis in Human Non-Hodgkin Lymphomas. Neoplasia.

[12] El-Kenawi AE, El-Remessy AB (2013). Angiogenesis inhibitors in cancer therapy: mechanistic perspective on classification and treatment rationales. Br J Pharmacol.

[13] Bao S, Wu Q, Sathornsumetee S, Hao Y, Li Z, Hjelmeland AB (2006). Stem cell-like glioma cells promote tumor angiogenesis through vascular endothelial growth factor. Cancer Res.

[14] Watnick RS (2012). The Role of the Tumor Microenvironment in Regulating Angiogenesis. Cold Spring Harb Perspect Med.

[15] DeFronzo RA, Goodman AM (1995). Efficacy of metformin in patients with non-insulin-dependent diabetes mellitus. The Multicenter Metformin Study Group. N Engl J Med.

[16] Ben Sahra I, Le Marchand-Brustel Y, Tanti JF, Bost F (2010). Metformin in cancer therapy: a new perspective for an old antidiabetic drug?. Mol Cancer Ther.

[17] Donadon V, Balbi M, Mas MD, Casarin P, Zanette G (2010). Metformin and reduced risk of hepatocellular carcinoma in diabetic patients with chronic liver disease. Liver Int.

[18] Chen TM, Lin CC, Huang PT, Wen CF (2011). Metformin associated with lower mortality in diabetic patients with early stage hepatocellular carcinoma after radiofrequency ablation. J Gastroenterol Hepatol.

[19] Lee MS, Hsu CC, Wahlqvist ML, Tsai HN, Chang YH, Huang YC (2011). Type 2 diabetes increases and metformin reduces total, colorectal, liver and pancreatic cancer incidences in Taiwanese: a representative population prospective cohort study of 800,000 individuals. BMC Cancer.

[20] Shackelford DB (2013). Unravelling the connection between metabolism and tumorigenesis through studies of the liver kinase B1 tumour suppressor. J Carcinog.

[21] Zhou J, Fan J, Wang Z, Wu ZQ, Qiu SJ, Huang XW (2006). Conversion to sirolimus immunosuppression in liver transplantation recipients with hepatocellular carcinoma: Report of an initial experience. World J Gastroenterol.

[22] Sahin F, Kannangai R, Adegbola O, Wang J, Su G, Torbenson M (2004). mTOR and P70 S6 kinase expression in primary liver neoplasms. Clin Cancer Res.

[23] Semela D, Piguet AC, Kolev M, Schmitter K, Hlushchuk R, Djonov V (2007). Vascular remodeling and antitumoral effects of mTOR inhibition in a rat model of hepatocellular carcinoma. J Hepatol.

[24] Forsythe JA, Jiang BH, Iyer NV, Agani F, Leung SW, Koos RD (1996). Activation of vascular endothelial growth factor gene transcription by hypoxia-inducible factor 1. Mol Cell Biol.

[25] Basu A, Contreras AG, Datta D, Flynn E, Zeng L, Cohen HT (2008). Overexpression of vascular endothelial growth factor and the development of post-transplantation cancer. Cancer Res.

[26] Daryani A, Sharif M, Dadimoghaddam Y, Souteh MB, Ahmadpour E, Khalilian A (2014). Determination of parasitic load in different tissues of murine toxoplasmosis after immunization by excretory-secretory antigens using Real time QPCR. Exp Parasitol.

[27] Yazdani Y, Farazmandfar T, Azadeh H, Zekavatian Z (2016). The prognostic effect of PTEN expression status in colorectal cancer development and evaluation of factors affecting it: miR-21 and promoter methylation. J Biomed Sci.

[28] Farazmandfar T, Haghshenas MR, Shahbazi M (2015). Inhibition of HIV-1 by a lentiviral vector with a novel tat-inducible expression system and a specific tropism to the target cells. Hum Gene Ther.

[29] Ebos JM, Lee CR, Cruz-Munoz W, Bjarnason GA, Christensen JG, Kerbel RS (2009). Accelerated metastasis after short-term treatment with a potent inhibitor of tumor angiogenesis. Cancer Cell.

[30] Dallaglio K, Bruno A, Cantelmo AR, Esposito AI, Ruggiero L, Orecchioni S (2014). Paradoxic effects of metformin on endothelial cells and angiogenesis. Carcinogenesis.

[31] Miyoshi H, Kato K, Iwama H, Maeda E, Sakamoto T, Fujita K, et al. Effect of the anti-diabetic drug metformin in hepatocellular carcinoma in vitro and in vivo. Int J Oncol 2013. 10.3892/ijo.2014.2419 24806290

[32] Gupta K, Kshirsagar S, Li W, Gui L, Ramakrishnan S, Gupta P (1999). VEGF prevents apoptosis of human microvascular endothelial cells via opposing effects on MAPK/ERK and SAPK/JNK signaling. Exp Cell Res.

[33] Wang W, Jia WD, Xu GL, Wang ZH, Li JS, Ma JL (2009). Antitumoral activity of rapamycin mediated through inhibition of HIF-1alpha and VEGF in hepatocellular carcinoma. Dig Dis Sci.

